# Body Satisfaction and Physical Appearance in Gender Dysphoria

**DOI:** 10.1007/s10508-015-0614-1

**Published:** 2015-10-16

**Authors:** Tim C. van de Grift, Peggy T. Cohen-Kettenis, Thomas D. Steensma, Griet De Cuypere, Hertha Richter-Appelt, Ira R. H. Haraldsen, Rieky E. G. Dikmans, Susanne C. Cerwenka, Baudewijntje P. C. Kreukels

**Affiliations:** Department of Medical Psychology, Center of Expertise on Gender Dysphoria, EMGO Institute for Health and Care Research, VU University Medical Center, P.O. Box 7057, 1007 MB Amsterdam, The Netherlands; Department of Plastic, Reconstructive & Hand Surgery, VU University Medical Center, Amsterdam, The Netherlands; Center of Sexology and Gender Problems, Ghent University Hospital, Ghent, Belgium; Department of Sex Research and Forensic Psychiatry, University Medical Center Hamburg-Eppendorf, Hamburg, Germany; Department of Neuropsychiatry and Psychosomatic Medicine, Division of Clinical Neuroscience, Rikshospitalet, Oslo, Norway

**Keywords:** Gender dysphoria, Body image, Appearance, Sexual orientation

## Abstract

Gender dysphoria (GD) is often accompanied by dissatisfaction with physical appearance and body image problems. The aim of this study was to compare body satisfaction with perceived appearance by others in various GD subgroups. Data collection was part of the European Network for the Investigation of Gender Incongruence. Between 2007 and 2012, 660 adults who fulfilled the criteria of the DSM-IV gender identity disorder diagnosis (1.31:1 male-to-female [MtF]:female-to-male [FtM] ratio) were included into the study. Data were collected before the start of clinical gender-confirming interventions. Sexual orientation was measured via a semi-structured interview whereas onset age was based on clinician report. Body satisfaction was assessed using the Body Image Scale. Congruence of appearance with the experienced gender was measured by means of a clinician rating. Overall, FtMs had a more positive body image than MtFs. Besides genital dissatisfaction, problem areas for MtFs included posture, face, and hair, whereas FtMs were mainly dissatisfied with hip and chest regions. Clinicians evaluated the physical appearance to be more congruent with the experienced gender in FtMs than in MtFs. Within the MtF group, those with early onset GD and an androphilic sexual orientation had appearances more in line with their gender identity. In conclusion, body image problems in GD go beyond sex characteristics only. An incongruent physical appearance may result in more difficult psychological adaptation and in more exposure to discrimination and stigmatization.

## Introduction

Gender dysphoria (GD) describes a status in which one experiences an incongruence between assigned and experienced gender. In line with societal and scientific changes, the development of the diagnostic criteria for GD has been subject to change since it first appeared as a diagnosis in the *Diagnostic and Statistical Manual of Mental Disorders* (American Psychiatric Association, 1980).

Although percentages of severe regret are as low as 1–2 % (Cohen-Kettenis & Gooren, 1999; Landen, Wålinder, Hambert, & Lundström, 1998), given the impact of gender affirming interventions, clinicians would like to be assisted in their treatment recommendations by adequate a priori assessment of factors that predict satisfaction with outcome. Some of the factors associated with dissatisfaction are physical build, incongruence with the new gender role, poor social support, and severe psychological morbidity (Gijs & Brewaeys, 2007). Body image and physical appearance are related to psychological well-being among GD individuals (Vocks, Stahn, Loenser, & Legenbauer, 2009). As the experienced incongruence between physique and gender identity/social role is the source of the dysphoria, GD has been conceptualized as a body image syndrome by some (e.g., Money, 1994). However, only a limited number of studies have specifically focused on body image in this group (Ålgars, Santtila, & Sandnabba, 2010; Bandini et al., 2013; Becker et al., 2015; Bodlund & Armelius, 1994; Fleming, MacGowan, Robinson, Spitz, & Salt, 1982; Kraemer, Delsignore, Schnyder, & Hepp, 2007; Lindgren & Pauly, 1975; Roback, Strassberg, McKee, & Cunningham, 1977; Vocks et al., 2009; Wolfradt & Neumann, 2001).

Body image is thought of as a person’s self-concept resulting from more than solely his or her visual self-image. It is conceptualized as consisting of attitudes, experiences, and perceptions pertaining to one’s physical appearance, based on self-observation and the reactions of others (see Cash & Pruzinsky, 2002). The degree of body (dis)satisfaction reflects one’s individual self-concept in relation to the social context. One may assume that a positive body image is a favorable prognostic factor of quality of life after transition (Bodlund & Armelius, 1994) whereas a negative body image may lead to lower quality of life due to lower self-esteem, poorer social functioning, and compensatory conditions, such as eating disorders (Ålgars et al., 2010; Bandini et al., 2013; Bodlund & Armelius, 1994; Vocks et al., 2009). We expect that, even in a population of individuals with GD who have serious body image problems, there is variation between individuals. In addition, clinically reported data on physical congruence with the experienced gender may inform us to what extent the source of body dissatisfaction can be attributed to differences in physical congruence between the individuals.

In the process of the development of the DSM-5 and the preparation of ICD-11, and as a result of the changing views on the relationship between GD and psychopathology, the GD diagnosis as well as the specification of certain subtypes have been topics of debate (Zucker et al., 2013). Sexual orientation and onset age of GD feelings are most frequently used to categorize people with GD.

With regard to sexual orientation, Blanchard, Clemmensen, and Steiner (1987) who proposed and studied a distinction between homosexual and non-homosexual individuals with GD, more recently also denoted with (non-)androphilia in male-to-females (MtFs) and (non-)gynephilia in female-to-males (FtMs) (Cerwenka et al., 2014; Lawrence, 2010). In some studies, it was found that, compared to non-androphilic MtFs, androphilic MtFs presented earlier for sex reassignment and reported more female identification in childhood (Lawrence, 2010; Smith, van Goozen, Kuiper, & Cohen-Kettenis, 2005a, 2005b). Non-androphilic MtFs, on the other hand, were more likely to have a history of sexual arousal by the image of themselves as a woman (Lawrence, 2010). As a result of criticism concerning the self-report bias and fluidity of sexual orientation, a subtyping based on onset age has been described (Lawrence, 2010; Smith et al., 2005b). Proponents of onset age-based subtyping argue that it better reflects the different developmental pathways (early onset [EO] and late onset [LO]) among the GD subtypes (Lawrence, 2010). Categorizing on the basis of onset age, however, is potentially complicated as well because of differences in puberty onset, biased recall, and subtype heterogeneity (Lawrence, 2010; Nieder et al., 2011). For example, within the LO MtF group, there are androphilic and gynephilic natal men.

Recently, Becker et al. (2015) addressed the differences in bodily satisfaction between GD individuals and controls. Specific information on subtype differences, in this case with regard to physical appearance and body image, can contribute to better clinical care. As both body satisfaction and therapeutic requests may be related to the age of onset and sexual preferences, knowledge on subtype differences may help to align gender reaffirming interventions to one’s personal situation.

### Aims

This study aimed to use the concepts of body image and physical appearance to provide a better understanding of GD, given their potential value in GD counseling. The main research objectives were (1) to describe body (dis)satisfaction and physical appearance with regard to onset age and sexual orientation in natal males and females with GD; (2) to examine the relationship between self-reported body satisfaction and clinician-reported physical appearance in individuals with GD.

## Method

### Participants

Of the 1019 applicants (MtF = 637 and FtM = 382), a total of 660 (64.7 %; MtF = 374 and FtM = 286) who received a GID diagnosis (American Psychiatric Association, 2000) and could be classified as EO or LO were included in the study. At the time of assessment, the DSM-5 was not published yet. Inclusion was based on a scoring sheet with GID diagnostic and onset age criteria (Nieder et al., 2011; see below as well). Of the excluded group (*n* = 359), 93 (9.1 %) did not fulfill all diagnostic criteria for GID, 103 fulfilled criteria for GID, but could not be assigned to either the early onset or late onset category (residual category), and 163 individuals who received a GID diagnosis had missing onset age data. There were no statistically significant differences in demographic characteristics (age and education) between the included and excluded groups. For 640 applicants, information on both onset age and sexual orientation was available. MtF applicants (*M* = 34.1 years, *SD* = 12.6 years) were significantly older compared to FtMs (*M* = 27.0 years, *SD* = 9.6 years, see Table 1). MtFs had significantly higher education than FtMs, *χ*^2^(2) = 12.51, *p* = .002. More than half of all included participants were diagnosed in Amsterdam, whereas the other clinics included 22.1 % (Ghent), 17.7 % (Hamburg), and 8.8 % (Oslo) of the subjects. The MtF to FtM ratio of the whole sample was 1.31:1. Ratios differed per country, which is in line with earlier research (Kreukels et al., 2012). As shown in Table 1, FtM applicants were significantly more likely to have (partially) transitioned than MtF applicants in private, *χ*^2^(2) = 26.35, *p* < .001 or work life, *χ*^2^(2) = 29.82, *p* < .001. Self-prescribed hormone use (self-report) prior to admission was significantly more common in MtFs (22.0 %) than in FtMs (7.3 %), *χ*^2^(1) = 27.71, *p* < .001. In MtF applicants, age of admission was significantly correlated with sexual orientation; younger MtFs were more likely to report androphilic orientation (*r* = .32, df = 365, *p* < .001) whereas this was not found in the FtM group (point biserial correlations; *r* = −.01, df = 271).

**Table 1:** Sample characteristics (applicants with formal GID diagnosis)

	MtF	FtM
	*n* = 374	*n* = 286
Mean age (in years; *SD*)^a^	34.1 (12.6)	27.0 (9.6)
Education		
Low (%)	79 (21.5)	66 (23.5)
Intermediate (%)	199 (54.1)	177 (63.0)
High (%)	90 (24.5)	38 (13.5)
Social role at admission (private)		
Experienced gender (%)	191 (52.6)	199 (72.6)
Variable (%)	125 (34.4)	54 (19.7)
Natal gender (%)	47 (12.9)	21 (7.7)
Social role at admission (work)		
Experienced gender (%)	126 (38.1)	134 (52.8)
Variable (%)	30 (9.1)	42 (16.5)
Natal gender (%)	175 (52.9)	78 (30.8)
Hormone use at admission (%)	81 (22.0)	16 (7.3)

Inclusions per center	*N*	%	MtF:FtM ratio
Amsterdam	339	51.4	1.63:1
Ghent	146	22.1	2.24:1
Hamburg	117	17.7	1:1.34
Oslo	58	8.8	1:3.46

Due to missing data, variable sums may not add up to the described number of participants

^a^ *t*(658) = 7.97, *p* < .001

### Procedure

Data collection was part of the European Network for the Investigation of Gender Incongruence (ENIGI) between January 2007 and October 2012. Individuals 17 years of age and older applying for sex reassigning interventions in Amsterdam (the Netherlands), Ghent (Belgium), Hamburg (Germany), and Oslo (Norway) were asked to participate. All data were collected during the diagnostic procedure before receiving any clinical gender-confirming medical interventions. For information on the ENIGI protocol, see Kreukels et al. (2012).

### Measures

Demographic data, information on social transitioning, previous hormone treatment, and sexual orientation were taken from a background interview (Kreukels et al., 2012).

The criteria of the formal GID diagnosis were scored on a self-constructed form, based on the DSM-IV-TR criteria (American Psychiatric Association, 2000). Based on a similar sheet, while using childhood diagnostic criteria, onset age was assessed. These forms were completed by the clinician at the end of the diagnostic phase. If both DSM-IV-TR core criteria of GID in childhood were fulfilled, individuals were categorized as EO (pre-pubertal “strong cross-gender identification” and “persistent discomfort about one’s assigned sex”). In case of (post-) pubertal onset of the GID (neither “strong cross-gender identification” nor “persistent discomfort about one’s assigned sex” before puberty were reported), individuals were classified as LO (Nieder et al., 2011). Individuals who fulfilled only one of the criteria in childhood were categorized in the residual group.

Sexual orientation was measured by one item from a semi-structured Background Interview (Kreukels et al., 2012) and classified according to the person’s experienced sexual attraction to others. Rating based on clinician-reported sexual orientation was strongly correlated to the self-reported measure (phi correlation; *ø* = .73, df = 616, *p* < .001). Results were scored on a Kinsey scale ranging from being exclusively attracted to one’s natal sex to being exclusively attracted to the other sex. Response categories for being attracted to transgenders or being asexual were added. Androphilia in MtFs and gynephilia in FtMs was defined as being attracted completely or primarily to one’s natal sex. Non-androphilic MtFs and non-gynephilic FtMs included all other options (having a bisexual attraction, an exclusive attraction to the other natal sex, an attraction to transgenders and asexuality).

Body image was measured by the Body Image Scale (BIS). It consists of 30 items to determine satisfaction with various body parts, rated on a 5-point scale of satisfaction ranging from very satisfied (1) to very dissatisfied (5) (Lindgren & Pauly, 1975). There are two versions of the scale: one for natal males and one for natal females. The BIS includes primary sex characteristics, secondary sex characteristics, and neutral (non sex-related) body parts. The BIS contains equivalent sex-specific genital body parts to enable MtF–FtM comparisons. Higher scores represent higher degrees of body dissatisfaction.

Lindgren and Pauly (1975) proposed a subscale analysis of the BIS, using the subscales primary sex characteristics, secondary sex characteristics, and neutral characteristics. However, these subscales do not allow for comparisons per body area. Therefore, an alternative clustering based on body areas within the BIS (see Table 2) was used. Cronbach’s alphas on the subscales for the sample are shown in Table 2.

**Table 2:** Body Image Scale subscales (Lindgren & Pauly, 1975)

Subscale	Items	Construct analysis
Lindgren and Pauly (1975)		
Primary sex characteristics	Body hair, breasts, facial hair, penis/clitoris, scrotum/vagina, and testicles/uterus	*α* = 0.65 6 items
Secondary sex characteristics	Appearance, arms, body movement, bottom, chest size, figure, hair, hips, muscles, thighs, upper arm muscles, voice, waist, and weight	*α* = 0.84 14 items
Neutral characteristics	Adam’s apple, chin, eye brows, face, feet, hands, height, legs, nose, and shoulders	*α* = 0.81 10 items
Body area subscales		
Social and hair items	Appearance, body hair, body movement, facial hair, hair, and voice	*α* = 0.72 6 items
Head and neck region	Adam’s apple, chin, eye brows, face, and nose	*α* = 0.74 5 items
Muscularity and posture	Arms, feet, hands, height, legs, muscles, shoulders, upper arm muscles, and weight	*α* = 0.79 9 items
Hip region	Bottom, figure, hips, thighs, and waist	α = 0.82 5 items
Chest region	Breasts and chest size	NA
Genitals	Penis/clitoris, scrotum/vagina, and testicles/uterus, and ovaries	*α* = 0.85 3 items

To measure physical appearance, the Physical Appearance Scale (PhAS) was used. This scale scores the observer’s appraisal of the masculinity/femininity of a person’s physical appearance, rated on a 5-point scale ranging from most congruent with the experienced gender (1) to most incongruent with the experienced gender (5) (Smith et al., 2005b). The scale contains 14 items, and scoring differs per natal sex. Higher scores represent a physical appearance that is less congruent with the experienced gender.

### Statistical Analysis

The degree of masculinity/femininity of the PhAS items was recoded, based on the person’s natal sex. Sexual orientation and onset age subgroups were compared with regard to overall scores, subscale scores, and scores on individual items of BIS and PhAS by means of one-way ANOVAs. These tests were carried out for the total group as well as for MtFs and FtMs separately. Within the natal sex groups, BIS scores were compared with regard to transition status, using independent *t*-tests. Bonferroni corrections were used to control for multiple comparisons. Bonferroni corrected *p* values were .0017 (.05/30) for BIS comparisons and .0036 (.05/14) for PhAS comparisons. Multiple stepwise linear regression analyses of sexual orientation and onset age predicting PhAS and BIS scores were performed. The correlation between sexual orientation and onset age was calculated using phi correlations. BIS subscale reliabilities were calculated by means of Cronbach’s alphas.

All analyses were repeated after excluding participants who were on hormonal therapy prior to admission, as hormones induce physical changes, and consequently may influence both femininity/masculinity of body parts and satisfaction with one’s physique.

## Results

### Distribution of Sexual Orientation and Onset Age Among Natal Males and Females

Within the MtF subgroup, the androphilic (*n* = 126)-non-androphilic (*n* = 241) ratio was 1:1.91 and the early (*n* = 190)-late onset (*n* = 177) ratio was 1.07:1. In the FtM group, the gynephilic (*n* = 219)-non-gynephilic (*n* = 54) ratio was 4.05:1 and the early (*n* = 230)-late onset (*n* = 43) ratio was 5.34:1 (Table 3). Sexual orientation and age of onset correlations were *ø* = .26 (MtFs; df = 365, *p* < .001), and *ø* = .21 (FtMs; df = 271, *p* < .001). This indicates a higher likelihood of androphilic sexual orientation in early onset MtFs and of gynephilic sexual orientation in early onset FtMs.

**Table 3:** Distribution of sexual orientation and onset age subgroups

	MtF		FtM	
	Androphilic	Non-androphilic	Gynephilic	Non-gynephilic
Early onset	88 (24.0%)	102 (27.8%)	193 (70.7%)	37 (13.6%)
Late onset	38 (10.4%)	139 (37.9%)	26 (9.5%)	17 (6.2%)

MtF; *χ* ^2^(1) = 25.09, *p* < .001; FtM; *χ* ^2^(1) = 12.55, *p* < .001

### Differences in Body Image and Physical Appearance Between the Natal Sexes

MtFs scored significantly higher than FtMs on the overall scores of both the BIS (ANOVAs; *M* = 101.27, *SD* = 15.66 vs. *M* = 96.27, *SD* = 14.93; *p* = .001) and the PhAS (ANOVAs; *M* = 42.28, *SD* = 9.55 vs. *M* = 39.18, *SD* = 7.00; *p* < .001) scales, indicating lower body satisfaction and a less congruent physical appearance with their experienced gender.

On the BIS items, MtFs reported highest dissatisfaction scores on the socially related body parts (such as voice), but also on their hair, their face and neck, and posture. FtMs, on the other hand, reported the highest discomfort with their breasts. Other reported areas of discomfort were the hip region and chest size. Dissatisfaction with the genitals was high in both groups, although MtFs tended to score higher on equivalent body parts (e.g., penis versus clitoris although not significant on all items). After Bonferroni correction for multiple comparisons, differences between MtFs and FtMs remained significant for most BIS items in ANOVA testing (Table 4).

**Table 4:** Body image and physical appearance scores in male-to-females versus female-to-males

	Self-reported (BIS)		Clinician-reported (PhAS)		Test characteristics	
	MtF *M* (*SD*)	FtM *M* (*SD*)	MtF *M* (*SD*)	FtM *M* (*SD*)	BIS^b^ *F* (df)	PhAS^b^ *F* (df)
Social and hair items						
Appearance	3.37 (1.13)	3.22 (1.07)	NA	NA	2.76 (1, 606)	NA
Body hair	4.33 (0.96)	3.37 (1.05)	NA	NA	139.45 (1, 606)***	NA
Body movement	2.99 (1.11)	2.58 (0.87)	2.44 (1.00)	2.01 (0.68)	25.19 (1, 613)***	35.94 (1, 610)***
Facial hair	4.66 (0.77)	3.67 (1.17)	3.10 (1.05)	3.20 (0.82)	154.81 (1, 597)***	1.61 (1, 612)
Hair	3.14 (1.37)	1.99 (0.77)	2.46 (1.24)	2.11 (0.85)	151.04 (1, 614)***	15.06 (1, 613)***
Speech	NA	NA	2.59 (1.04)	2.28 (0.82)	NA	15.67 (1, 611)***
Voice	3.99 (1.09)	3.86 (1.12)	3.22 (1.10)	2.92 (0.98)	1.91 (1, 611)	11.77 (1, 614)**
Head and neck region						
Adam’s apple	3.62 (3.62)	3.25 (1.04)	3.40 (0.84)	3.24 (0.71)	13.01 (1, 564) ***	5.98 (1, 599)*
Chin	2.98 (1.04)	2.25 (0.90)	3.16 (0.81)	2.88 (0.72)	83.99 (1, 613)***	19.83 (1, 613)***
Eye brows	2.86 (1.11)	2.18 (0.81)	NA	NA	69.87 (1, 612)***	NA
Face	3.27 (1.08)	2.68 (0.99)	NA	NA	47.27 (1, 611)***	NA
Jaw	NA	NA	3.21 (0.82)	2.89 (0.71)	NA	25.08 (1, 611)***
Nose	3.03 (1.21)	2.02 (0.78)	3.24 (0.85)	2.85 (0.68)	142.22 (1, 613)***	39.59 (1, 612)***
Skin	NA	NA	2.98 (0.89)	3.06 (0.73)	NA	1.73 (1, 613)
Muscularity and posture						
Arms	2.64 (0.90)	2.58 (0.98)	NA	NA	< 1 (1, 616)	NA
Feet^a^	3.14 (1.13)	2.52 (0.90)	3.27 (0.95)	3.07 (0.78)	55.24 (1, 616)***	7.40 (1, 611)**
Hands^a^	2.93 (1.11)	2.43 (1.00)	3.27 (0.95)	3.07 (0.78)	33.38 (1, 612)***	7.40 (1, 611)**
Height	2.62 (1.14)	3.02 (1.13)	3.16 (1.03)	3.25 (0.92)	18.66 (1, 610)***	1.21 (1, 615)
Legs/calves	2.54 (0.97)	2.35 (0.96)	NA	NA	5.91 (1, 607)*	NA
Muscles	3.01 (0.98)	3.12 (1.14)	3.06 (0.80)	2.81 (0.77)	1.55 (1, 610)	14.79 (1, 611)**
Shoulders	2.92 (1.04)	2.35 (1.05)	NA	NA	45.64 (1, 616)***	NA
Upper arm muscles	3.20 (1.02)	3.20 (1.14)	NA	NA	<1 (1, 597)	NA
Weight	2.88 (1.18)	3.03 (1.14)	NA	NA	2.680 (1, 612)	NA
Hip region						
Bottom	2.89 (1.08)	3.32 (1.06)	NA	NA	24.54 (1, 615)***	NA
Figure	3.13 (1.14)	3.56 (1.06)	3.00 (1.06)	2.76 (0.96)	22.75 (1, 610)***	7.89 (1, 613)**
Hips	3.25 (1.11)	3.62 (1.14)	NA	NA	16.31 (1, 615)***	NA
Thighs	2.75 (1.03)	3.33 (1.10)	NA	NA	44.74 (1, 607)***	NA
Waist	3.09 (1.12)	3.63 (1.03)	NA	NA	37.07 (1, 600)***	NA
Chest region						
Breasts	4.20 (1.05)	4.81 (0.60)	NA	NA	69.45 (1, 601)***	NA
Chest size	3.54 (1.10)	4.02 (1.13)	NA	NA	26.92 (1, 595)***	NA
Genitals						
Penis/clitoris	4.55 (0.82)	4.28 (1.02)	NA	NA	12.55 (1, 591)***	NA
Scrotum/vagina	4.62 (0.68)	4.54 (0.81)	NA	NA	1.46 (1, 593)	NA
Testicles/ovary	4.64 (0.68)	4.61 (0.77)	NA	NA	<1 (1, 590)	NA
Overall	101.27 (15.66)	96.27 (14.93)	42.28 (9.55)	39.18 (7.00)	11.01 (1, 435)**	18.94 (1, 593)***

One-way ANOVA; Bonferroni corrected: PhAS .05/14 = .0036; BIS .05/30 = .0017; *NA* the item is not applicable to this scale

^a^Combined item in PhAS

^b^* *p* < .05, ** *p* < .01, *** *p* < .001

Clinicians assessed FtMs’ appearance as more congruent with the experienced gender than MtFs’ appearance. MtFs’ appearance was less congruent with the experienced gender regarding motor movement, speech and voice, hair, facial features, and muscularity. All significant differences between MtFs and FtMs in PhAS items, except for Adam’s apple, feet/hands and figure, remained significant after Bonferroni correction.

When comparing the outcomes of the BIS and PhAS (sub)scores, clinicians and applicants reports generally showed similar patterns. This implies that the physical characteristics related to higher dissatisfaction were mostly the ones also considered less congruent by clinicians. The only item in which self-report conflicted with clinician report was the figure (clinicians reported FtMs more congruent although they reported to be more dissatisfied with their figure).

No major differences in the overall and subscale scores were observed after excluding participants on prior hormonal treatment (*n* = 97). Only the BIS Adam’s apple item was no longer significantly different between the MtF and FtM subgroups (ANOVA; *p* = .002 after Bonferroni correction). When comparing participants with and without prior hormone use, overall physical congruence with the experienced gender was significantly lower in people who did not receive hormonal treatment (*M* = 41.27) compared to the ones who did (*M* = 39.77, *p* = .006). No significant difference between overall BIS scores was found (all ANOVAs).

### Differences in Body Image and Physical Appearance Between Sexual Orientation and Onset Age Subgroups Among MtFs and FtMs

**Fig. 1:**
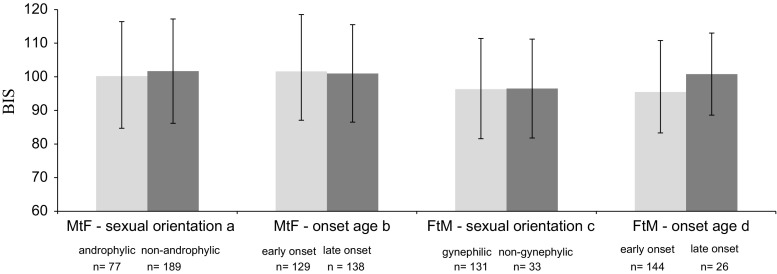
Total Body Image Scale scores in male-to-female and female-to-male sexual orientation and onset age subgroups. One-way ANOVA, absolute range = 30 (most satisfied)–150 (most dissatisfied). ^a^ *F* < 1, df = 1, 264; ^b^ *F* < 1, df = 1, 265; ^c^ *F* < 1, df = 1, 162; ^d^ *F* < 1, df = 1, 168

With regard to overall body satisfaction (i.e., BIS scores), no significant differences between sexual orientation and onset age subgroups were found in both natal sexes. The only trend was the relatively highly reported body dissatisfaction of LO FtMs (approaching MtF levels) compared to their EO counterparts (*p* = .095; see Fig. 1). Excluding participants who used hormonal therapy prior to admission did not change these findings.

**Fig. 2:**
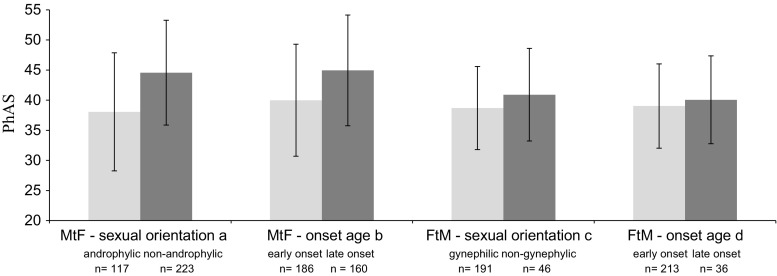
Total Physical Appearance Scale scores per subgroup in male-to-female and female-to-male sexual orientation and onset age subgroups. One-way ANOVA, absolute range = 14 (most congruent with experienced gender)–70 (least congruent with experienced gender). ^a^ *F* = 39.29, df = 1, 338, *p* < .001; ^b^ *F* = 24.69, df = 1, 344, *p* < .001; ^c^ *F* = 3.708, df = 1, 235, *p* = .055; ^d^ *F* < 1, df = 1, 247

Concerning the overall congruence of physical appearance (i.e., PhAS scores), non-androphilic MtFs were considered significantly less feminine than androphilic MtFs (*p* < .001). Similarly, LO MtFs scored significantly less feminine than EO MtFs (*p* < .001) in ANOVA testing. In FtMs, no statistically significant subgroup differences were found. Nevertheless, gynephilic and EO applicants tended to score somewhat more congruent with the experienced gender (see Fig. 2). Repeating the analyses while excluding participants who used hormonal therapy prior to admission resulted in similar findings.

Regression analysis showed that, in the MtF subgroup, satisfaction with body parts of social relevance and hair was predicted by sexual orientation (see Table 5). Furthermore, overall reported physical congruence was predicted by both sexual orientation and onset age. In the sample as a whole, sexual orientation and onset age were both weak predictors of body image and physical appearance, although sexual orientation was a somewhat stronger predictor than onset age. This suggests that MtFs with early onset gender dysphoria and androphilic sexual orientation more often have a more satisfactory body image and physical appearance congruent with their gender identity. No significant predictors for body image and physical appearance scores were found in the FtM applicants.

**Table 5:** Results of regression analyses for physical appearance and body image with sexual orientation and onset age as predictors

	Predictors^a,c^	*β* ^b,d^
Whole sample		
BIS sum	Sexual orientation	.10*
BIS social and hair items	Sexual orientation	.28***
	Onset age	−.11**
BIS chest region	Sexual orientation	−.16***
	Onset age	.09*
PhAS sum	Sexual orientation	.26***
	Onset age	.16***
MtFs		
BIS social and hair items	Sexual orientation	.18**
BIS genitals	Onset age	−.13*
PhAS sum	Sexual orientation	.27***
	Onset age	.20***

^a^Sexual orientation labels: 1 = androphilic (MtFs) or gynephilic (FtMs), 2 = non-androphilic (MtFs) or non-gynephilic (FtMs); onset age labels: 1 = early onset, 2 = late onset

^b^Higher PhAS corresponds with less physical congruence with the experienced gender; higher BIS scores represent higher degree of body dissatisfaction

^c^Phi correlation sexual orientation and onset age; *ø* = .36, df = 638, *p* < .001

^d^* *p* < .05, ** *p* < .01, *** *p* < .001

*BIS* Body Image Scale, *PhAS* Physical Appearance Scale

### Social Transition and Body Image

In MtFs, social transition in private life at clinical admission corresponded with lower BIS scores (*M* = 107.97, *SD* = 13.84 vs. *M* = 96.39, *SD* = 16.07 for socially transitioned; *p* < .001), indicating lower body dissatisfaction in this group. The same was found for social transition at work (*M* = 106.02, *SD* = 13.53 vs. *M* = 94.50, *SD* = 15.27 for socially transitioned; *p* < .001). In the FtM group, no such differences were found when performing ANOVAs.

## Discussion

The objective of this study was to assess self-reported body (dis)satisfaction and physical appearance as evaluated by clinicians in relation to various subgroups of individuals with GD before the start of medical treatment.

As expected, individuals reported the highest degree of dissatisfaction with their primary and secondary sex characteristics, but body dissatisfaction in GD appeared to go beyond this kind of sex-anatomically related dysphoria. The findings on primary and secondary sex characteristics were generally in line with earlier research (Ålgars et al., 2010; Bandini et al., 2013; Bodlund & Armelius, 1994; Fleming et al., 1982; Kraemer et al., 2007; Vocks et al., 2009; Wolfradt & Neumann, 2001). Our data showed higher overall scores on both the BIS and the PhAS in MtFs, compared to FtMs, indicating less body satisfaction and a physical appearance that was less congruent with the experienced gender. As described earlier, body image is often conceptualized as one’s self-concept of physique in relation to the social context (Cash & Pruzinsky, 2002). The source of the observed differences in body satisfaction between the groups, therefore, may be found in both physical characteristics and psychosocial characteristics.

Significantly more FtMs lived (partially) in their experienced gender role, compared to MtFs. Transition in both private and work life before they entered the clinic corresponded with significantly lower reported body dissatisfaction. Therefore, the difference between the sexes in body (dis)satisfaction may be related to the difference in social transitioning between the groups: FtMs are more satisfied with their body and this may be due to more frequent social transition. In society, the masculine role of FtMs is generally more accepted than the female role of MtFs, making social transition for the first group easier. Living in the social role of the experienced gender may contribute to a more positive attitude toward one’s body. On the other hand, the ones who already have a more positive body image may also be the ones that transitioned earlier.

Other factors that differ between the sexes and that may explain differences in body (dis)satisfaction between these groups are prior hormone use and age. We could, however, not confirm a relation between prior hormone use or age and the degree of body (dis)satisfaction. Although (self-)administration of hormones is expected to influence the congruence of physical characteristics with the experienced gender, the results may have been unsatisfactory, because these individuals applied to a clinic to receive further gender-confirming treatment. In addition, hormones may not have been used long enough or in suboptimal doses to induce the desired physical changes.

Clinicians judged FtMs as more physically congruent with the experienced gender than MtFs on all listed body items. As MtF applicants were older than FtMs, and age was significantly correlated with higher physical incongruence scores, this may explain some of the difference between the natal sexes. In addition, social transition may not only favor body satisfaction directly, but also influence the social evaluation of a person’s physical characteristics, and significantly more FtMs lived (partially) in their experienced gender role, compared to MtFs. Finally, sex differences in physical appearance and body satisfaction may be explained by the construction of gender as described by Kessler and McKenna (1978). The attribution of gender primarily depends on the existence or absence of male traits (e.g., physical masculinization). Masculine body characteristics (e.g., hair growth, facial characteristics) are often more difficult to mask and, therefore, it may be more difficult for MtFs to present themselves in a feminine way than vice versa. A similar argument for social transition and body image may be followed here for social transition and physical appearance. Social transition may be easier for FtMs, because their appearance is more easily aligned to their experienced gender. In addition, people who have already transitioned might be more easily perceived as their experienced gender than those who have not.

The observation that FtMs were perceived as more congruent with their experienced gender, even without (hormonal) treatment, may also be indicative of the social attitudes toward bodily masculinity and femininity. For MtFs, pronounced features, such as jaw line or facial hair growth, may impede their feminine appearance. These body attributes, which are most difficult to hide, are the ones with the highest dissatisfaction scores, when compared to the other sex. The different areas of dissatisfaction for the natal sexes could also be the result of a difference of importance which is attributed to this item in personal and societal standards (e.g., masculine mesomorphic standard), how this body item impacts social interaction, and if it can be influenced via modifying techniques (e.g., such as make-up, clothing, surgery, or weight loss).

With regard to reported total body (dis)satisfaction, no subtype differences were found within the MtF and FtM groups. Clinicians, however, viewed the physical appearance of applicants with a sexual preference for their natal sex (i.e., androphilic MtFs and gynephilic FtMs) and with an early onset more congruent with their experienced gender. Their sexual preference and relational experiences may have steered androphilic MtFs and gynephilic FtMs toward presenting their appearance in a more congruent way. In contrast, non-androphilic MtFs and non-gynephilic FtMs may have had “heterosexual” relationships prior to admission, in which they may have been more likely to present their physique in a way that corresponded with social norms of the natal sex, rather than of the experienced gender (Cerwenka et al., 2014). In case of LO gender dysphoric people, this may be related to the fact that cross-gender identification and presentation became more present at a later age. Findings on physical differences between sexual orientation subtypes (Blanchard et al., 1987), such as lower body weight of androphilic MtFs, have not been replicated (Smith et al., 2005b). Moreover, sexual orientation is described to be fluid over time (Cohen-Kettenis & Pfäfflin, 2010). Therefore, an explanation for the perceived subtype differences in physical congruence may be more likely found in differences in relational role and the use of body part modifying techniques between the sexual orientation subgroups. Individuals who are enabled to live in the social role of their experienced gender within their relationships may feel more empowered to express this role socially through clothing, hairstyle, make-up, and physical behavior.

An explanation for the more congruent physical appearance of the EO versus the LO applicants may be found in their younger age. As mentioned before, younger age was found to be associated with a more congruent physical appearance with the experienced gender. In addition, it may be easier to physically “pass” as the experienced gender when transitioning earlier in life, resulting in higher chance of finding a partner from the preferred gender role and developing a more satisfactory self-image.

In relation to physical appearance, one’s sexual orientation may also be informative on possible membership of a certain subculture. The male homosexual subculture is known to have high standards on physical appearance whereas the lesbian subculture is more tolerant toward diversity in appearance (Ålgars et al., 2010; Morrison, Morrison, & Sager, 2004; Vocks et al., 2009). Applying high bodily standards to oneself may increase the likelihood of body dissatisfaction. Sexual orientation and relational functioning may also influence one’s treatment preferences (Cerwenka et al., 2014); sexuality could be a decisive factor in choosing for a phalloplasty or characteristics of the neovagina (such as depth).

The relationship between sexual orientation and onset age remains a topic of debate (Lawrence, 2010). Recently, the sub workgroup on the DSM-5 classification concluded that clinical decisions are currently no longer based on the sexual orientation classification (Zucker et al., 2013). Onset age and sexual orientation correlate as low as ø = .26 (MtFs) and .21 (FtMs), a finding in line with earlier research (Lawrence, 2010). Therefore, one cannot be substituted for the other. Although both variables appeared to be weak predictors of body (dis)satisfaction and of clinician-viewed physical congruence, sexual orientation appeared to be a stronger predictor of physical appearance, and (aspects of) body image than onset age. Therefore, information on sexual orientation, acknowledging the shortcomings of this concept, may contribute to a more focused counseling in some individuals when it comes to body changing interventions. Gender role in previous relationships and the assumed impact of medical interventions should be subject of counseling. Also, sexual behavior should be considered when choosing gender affirming interventions (e.g., possibility of vaginal penetration).

### Limitations

The current study was limited by the self-report character of sexual orientation, onset age, and BIS. As data were collected during the diagnostic phase, individuals might have responded in a socially desirable way to receive a diagnosis and, therefore, treatment. Furthermore, the assessment of physical appearance was done by only one clinician. However, earlier data published on this scale found inter-observer correlation coefficients ranging from .68 to .79 for the individual items (Smith et al., 2005a).

Data on the BIS and PhAS scales were collected at different moments of the diagnostic phase; data on body image were collected at the beginning of the diagnostic procedure, whereas data on physical appearance were collected later on in the diagnostic process. Finally, as mentioned earlier, the concept of sexual orientation has its limitations. In the GD population, sexual orientation may be subject to change over the course of transition, perhaps even more than in non-GD populations (Cohen-Kettenis & Pfäfflin, 2010). Therefore, the conclusions of this study regarding this concept merely apply to the phase before clinical interventions and the diagnostic considerations made at that point. Also, in this study sexual orientation was coded as a dichotomous measure whereas actual sexual orientation may be viewed on a continuum. The classification based on the onset of gender dysphoria is limited by the fact that some people could not be categorized as early or late onset (i.e., the residual group).

### Conclusion

Body image problems in GD go beyond sex characteristics and congruence of physical appearance only. As body dissatisfaction may be indicative of one’s ability to adapt to one’s body and of (hidden) clinical expectations, it seems a valuable target of counseling at admission. Particularly, individuals with low body satisfaction extending beyond sex characteristics only should receive special attention. Information on sexual orientation may be informative as it may have an impact on preferences for body-related interventions. Ultimately, more effective counseling should make individuals more resilient during transition and medical interventions. As slightly different patterns of clinician-reported PhAS and self-reported BIS scores were observed, one should be aware of the potential bias between external and internal interpretation of physique. A congruent appearance does not necessarily imply a positive body image. Therefore, clinicians should remain sensitive to potential body image issues in all applicants, not specifically in the group who they expect it to have.
